# Role of urothelial cells in BCG immunotherapy for superficial bladder cancer

**DOI:** 10.1038/sj.bjc.6602026

**Published:** 2004-07-13

**Authors:** R F M Bevers, K-H Kurth, D H J Schamhart

**Affiliations:** 1 Department of Urology, Leiden University Medical Center J3-P, PB 9600, 2300 RC Leiden, The Netherlands; 2 Department of Urology, Academic Medical Center G-4, Meibergdreef 9, 1105 AZ Amsterdam, The Netherlands

**Keywords:** Bacillus Calmette-Guérin, bladder cancer, immunotherapy, cytokine

## Abstract

Intravesical instillation of Bacillus Calmette-Guérin (BCG) is used for the treatment of superficial bladder cancer, both to reduce the recurrence rate of bladder tumour and to diminish the risk of progression. Since its first therapeutic application in 1976, major research efforts have been directed to decipher the exact mechanism of action of the BCG-associated antitumour effect. Bacillus Calmette-Guérin causes an extensive local inflammatory reaction in the bladder wall. Of this, the massive appearance of cytokines in the urine of BCG-treated patients stands out. Activated lymphocytes and macrophages are the most likely sources of these cytokines, but at present other cellular sources such as urothelial tumour cells cannot be ruled out. Bacillus Calmette-Guérin is internalised and processed both by professional antigen-presenting cells and urothelial tumour cells, resulting in an altered gene expression of these cells that accumulates in the presentation of BCG antigens and secretion of particular cytokines.

Bladder cancer is detected in 75% of patients at an early, superficial stage. The majority of the superficial urothelial cell carcinomas (TCCs) consists of papillary bladder carcinomas (Figure 1A

**Figure 1:**
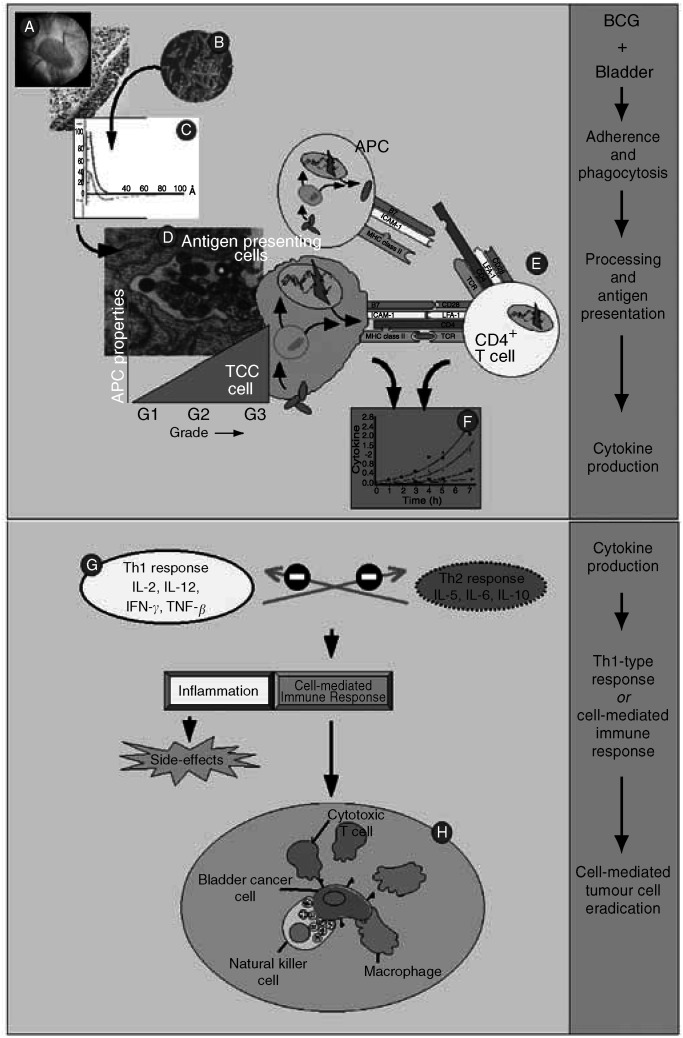
Simplified scheme of the supposed mechanism of action of BCG in tumour cell eradication. After its instillation in the bladder (**A**), BCG (**B**) accumulates near the bladder wall, followed by adherence and passage through the GAG layer of the bladder wall (**C**). Bacillus Calmette-Guérin is internalised and processed by professional antigen-presenting cells (APCs) and (high-grade) tumour cells (**D**), and BCG antigens are presented to CD4^+^ T cells (**E**). Depending on various conditions, this results in the local synthesis of a particular set of cytokines, known as the Th1-type or cell-mediated immune response (**F**, **G**). The Th1 cytokine profile enables recruitment and maturation of cytotoxic effector cells. No definite statements can be made yet about the actual effector cell(s), but a key role for NK cells in tumour cell killing has been proposed (**H**).

), while the remaining (10%) is called carcinoma *in situ* (CIS), a high-grade diffuse surface-spreading lesions. The standard treatment is trans-urethral resection (TUR) followed by intravesical instillation with adjuvant, chemo- or immunotherapeutic drugs (Kurth *et al*, 2000). For treatment of the surgically nonaccessible CIS, these latter modalities are the only bladder sparing options available. Although the therapeutic efficacy is well recognised, a high recurrence rate of 60–70% and a progression rate of 15% dictate the need for lifelong follow-up and treatment.

Adjuvant agents were introduced to reduce the risk for recurrence and progression of TCC. Recent, comparative trials indicate that immunotherapy with Bacillus Calmette-Guérin (BCG; Figure 1B) is superior to chemotherapy in patients with intermediate to high risk for recurrence. In these patients, the benefits of treatment outweigh the burden of side effects. Moreover, BCG seems to exert a better antitumour effect in CIS and high-grade cancer (Melekos *et al*, 1993; Kurth *et al*, 2000).

Bacillus Calmette-Guérin, an attenuated strain of *Mycobacterium bovis*, was developed by Calmette and Guérin with the intention to generate a vaccine against tuberculosis. The clinical efficacy of the commercially available strains of BCG, such as Connaught and Tice, appears to be comparable (Witjes *et al*, 1996; Champetier *et al*, 2000).

Since the first report of intravesical use of BCG in 1976, investigators try to understand the working mechanism of BCG as an antitumour modality. Both BCG treatment regimen and dose are historically determined. Arbitrarily, BCG therapy consists of a single course of six weekly intravesical instillations. Extension of BCG treatment (maintenance immunotherapy) is used to increase efficacy. Despite its success, 30–50% of patients either fail to respond or relapse within the first 5 years of treatment. Unfortunately, BCG, a viable, living organism, can cause infections resulting in side effects ranging from bothersome cystitis in the majority of patients to sepsis eventually leading to death in rare cases.

In order to reduce the side effects of BCG and to improve efficacy, interesting approaches are developed, such as the application of a cell wall–DNA complex (MCC) of *Mycobacterium phlei,* which was effective in patients who failed BCG therapy (Morales *et al*, 2001), genetically engineered BCG secreting relevant cytokines such as human lL-2 (Yamada *et al*, 2000), or treatment schedules consisting of three times viable BCG and three subsequent instillations with killed BCG (De Boer *et al*, 2003).

This review presents an overview of the current state of ‘BCG research’. Special attention will be paid to the role of the urothelial cells in the cascade of events leading to BCG-associated tumour cell clearance. As an underdeveloped field of research, the significance of an interaction between potentially hostile bacteria and epithelial cells is a relatively unknown aspect of the host immune-defense system. Better knowledge about this interaction might open new avenues for improvement of the BCG immunotherapy.

For an actual overview on the practical, clinical aspects of the immunotherapy for TCC with BCG, the reader is referred to various, recently published papers (Kurth *et al*, 2000).

## DETAILS OF THE MODE OF ACTION OF BCG

Nowadays, it is generally assumed that the BCG-induced antitumour activity is critically dominated by a local nonspecific immunological reaction reflecting the activity of immunocompetent cells (Alexandroff *et al*, 1999). It should be realised however that preceding the activation of the local immune system, some possibly rate-limiting events need to be executed. The sequential order of these initial events has been addressed only to a limited extent.

### Interaction of BCG with the bladder wall

Interaction of BCG with the luminal surface of the bladder is the first step for BCG to accomplish. Accumulation of BCG near the bladder wall and adherence may be limiting processes for an adequate, clinical response. Suboptimal binding of BCG may explain the absence of clinical response in a subgroup of patients (Ratliff *et al*, 1987; Schamhart *et al*, 1994). Experimental modulation of BCG attachment affects BCG-induced response in animal models (Hudson *et al*, 1991). Systematic analysis of the interaction of BCG with the bladder wall has not been accomplished, probably due to poor recognition of the biological and physicochemical processes involved. The process of interaction should be divided into nonspecific, physicochemical and specific, receptor–ligand-mediated events.

#### Physicochemical interaction

The luminal side of the bladder is covered with a layer of hydrophilic, highly sulphated glycosaminoglycans (GAGs). This GAG layer protects the bladder from toxic compounds and microorganisms. Both the GAG layer and the BCG cell wall are highly negatively charged (the *zeta* potentials). These conditions prescribe that BCG bacteria accumulate *without adherence*, at a close docking distance (70–100 Å) to the bladder wall (Figure 1C) (Schamhart *et al*, 1994). In addition to this *reversible* adsorption, physicochemical considerations predict a low probability of *irreversible* adherence of BCG to the bladder wall, due to the high electrostatic, repellent force between the respective surfaces. The observed, in animals, low abundance of BCG adherence to the uninjured bladder wall and the dependency of BCG adherence to diluent properties (pH, salt concentration) seem to be in accord with these theoretical considerations (Ratliff *et al*, 1987; Hudson *et al*, 1991; Teppema *et al*, 1992). Damage of the GAG layer and urothelium may lower the negative charge of the bladder wall, leading to an increased BCG docking and adherence, as observed in a murine BCG model after electrocautery damage of the bladder (Ratliff *et al*, 1987).

#### Specific, receptor–ligand-mediated events

In addition to nonspecific interaction, more specific mechanisms seem to be involved in BCG adherence. A crucial binding of BCG to fibronectin (FN) in the bladder mucosa has been postulated (Kavoussi *et al*, 1990). Fibronectin is part of the extracellular matrix, is equally distributed on normal and malignant urothelium, and a soluble form can be found in urine. Binding of BCG to the murine bladder was impaired with anti-FN antibodies or addition of soluble FN (Kavoussi *et al*, 1990). Furthermore, BCG bacteria possess a receptor with high affinity for the collagen domain of FN, the fibronectin attachment protein (FAP). These data and the observation that the clinical effect of BCG therapy is related to the degree of FN expression on normal mucosa suggest a specific FN-mediated adherence of BCG to the bladder wall. However, these experimental, animal data were largely obtained with preinjured bladders, contradictory to the clinical situation, and a fortuitous relationship between the efficacy of BCG therapy and fibronectin has been suggested (Bevers *et al*, 1998, 2000).

### Internalisation of BCG and phenotypical alterations of urothelial cells

#### Internalisation of BCG in urothelial cells

Several reports show that urothelial cells are capable of internalisation of BCG (Bevers *et al*, 1998, 2000; Durek *et al*, 1999). Bacillus Calmette-Guérin internalisation *in vitro* is time and dose dependent and can already be demonstrated after a 15-min incubation period. In a spheroid 3-D model, internalised BCG was found four cell layers deep, whereas normal urothelial cells in this system did not internalise BCG (Durek *et al*, 1999). Guinea pig studies showed no adherent or internalised BCG in normal urothelial cells (Teppema *et al*, 1992). In malignant cells, BCG internalisation appeared to be cell differentiation dependent. Contrary to well-differentiated (G1) bladder tumour cell lines, poorly differentiated (G3) cell lines exhibit significant internalisation of BCG (Bevers *et al*, 1998). Clinically, these data relate well to observations that show a better response to BCG treatment of high-grade compared to low-grade tumours (Table 1

**Table 1:** Recurrence rate/100 patients-months in patients with superficial bladder cancer, following TUR with and without adjuvant intravesical BCG instillations

**Treatment**	**Grade 1**		**Grade 2**		**Grade 3**	
TUR	1.5	NS	3.67	*P*<0.01	20.83	*P*<0.002
TUR+BCG	1.18		1.02		1.26	

a Data from Melekos *et al* (1993); NS=not significant.

) (Melekos *et al*, 1993).

Knowledge about the mechanism of BCG internalisation is scarce. Fibronectin is suggested to act as a bridging molecule, binding both to urothelial cells and BCG. Urothelial cells express an integrin (*α*5*β*1 receptor) with affinity for FN (Zhao *et al*, 2000). Pretreatment of T24 cells, a human TCC cell line, with anti-FN or anti-*α*5*β*1-receptor antibodies resulted in an impaired BCG attachment to and internalisation in these cells. However, other investigators could not inhibit BCG internalisation with anti-FN antibodies (Schneider *et al*, 1994; Bevers *et al*, 2000). The current available data do not suggest a mandatory role of FN in BCG internalisation. It seems that BCG internalisation is mediated by additional molecule(s), possibly coexpressed with FN, such as heparan sulphate-containing proteoglycans interacting with the mycobacterial heparin-binding haemagglutinin adhesin (HBHA) (Schneider *et al*, 1994; Bevers *et al*, 2000).

Regardless of the exact mechanism of internalisation, intracellular BCG is transported within phagosomes. These fuse with lysosomes to form phagolysosomes. Recent evidence shows that intracellular, live BCG can interfere with this phagolysosome formation (Maksymowych and Kane, 2000). However, only a small fraction of commercially available BCG preparations consists of live BCG. Therefore, most BCG particles will be degraded, and mycobacterial glycoproteins and lipoproteins are transported to the cell surface (Neyrolles *et al*, 2001).

Patient studies, analysing bladder washings after BCG instillations, revealed vigorous phagocytosis of BCG by leucocytes. However, concerning internalised BCG in urothelial cells, conflicting results are reported (Teppema *et al*, 1992). Accepting the inability of normal urothelial cells to internalise BCG, these conflicting observations may be explained by the presence or absence of residual urinary tumour cells in the limited number of patients included in both studies.

In summary, BCG particles can be internalised and processed by residual, especially high-grade tumour cells. As a consequence, the possibility that BCG introduces phenotypical alterations of TCC cells that affect tumoricidal effector mechanisms has been a subject of study. Several effects, ranging from a direct antiproliferative/cytotoxic effect of BCG, to a role in the initiation and/or modulation of the host immune response, and to an increase of susceptibility of tumour cells, have been proposed.

### Cytotoxic effects of BCG on BCG-internalising urothelial cells

*In vitro* studies with human TCC cell lines show that BCG exerts cytolytic, antiproliferative and antimotility effects. The inhibitory effects on cell proliferation were most pronounced in highly dedifferentiated cell lines. Today, the causal mechanisms are unknown. Internalised BCG increased the production of cytotoxic nitric oxide (NO) in TCC cells. Patients treated with BCG showed an augmented bladder NO production and an upregulation of urothelial-associated nitric oxide synthase (Jansson *et al*, 1998). NO, at high concentration, may cause DNA damage, and cytostatic and cytotoxic effects. Possible accumulation of DNA damage may be related to the observation that, contrary to nontreated patients, urothelial cells of BCG-treated patients express regulatory genes related to DNA repair, knowingly wild-type p53 and P21^waf1/cip1^ (Fontana *et al*, 1999). Whether these observations represent an indirect or direct cytotoxic effect of BCG *in situ* remains unclear.

### Bacillus Calmette-Guérin-internalising urothelial cells and the initiation/modulation of the immune response

Activation of the host immune system has been considered an exclusive characteristic of professional antigen-presenting cells (APCs), like dendritic cells and macrophages. New insights support a role for the interaction of (airway)epithelial cells and bacteria in the initiation of the immunological cascade (Levine, 1995; Neyrolles *et al*, 2001). Bacillus Calmette-Guérin-treated urothelial tumour cells are now considered as active participants in the cytokine-mediated initiation and/or regulation of the early immune response (Alexandroff *et al*, 1999).

#### The initial immune response to BCG

The local immune response to bacteria, including live BCG, is complex, but the presentation of bacterial antigens by APC to T-helper cells is the pivotal interaction (Alexandroff *et al*, 1999). Derived from an extensive series of papers, it appears that, after internalisation and processing by professional APC, processed BCG antigen(s) become associated with MHC class II molecules (Figure 1D). The antigen–MHC class II complex is expressed at the cell surface to be recognised by CD4^+^ T-helper lymphocytes via the T-cell receptor (TCR) molecule (Figure 1E). Along with this binding, the interaction between the APC and CD4^+^ T cell is only fully accomplished by an additional series of costimulatory, but essential, interacting molecules. Among others, binding of CD4 to MHC class II, lymphocyte function-associated antigen-3 (LFA-3) to intercellular adhesion molecule-1 (ICAM-1) and CD28 (CD4^+^ cell) to B7 enhances the conjugation of the cells and promotes T-cell activation signals. Soluble cytokines, such as IL-2, IL-6 and IFN-*γ*, provide generation of APC and T cells and ‘fine-tuning’.

Depending on many nonspecific factors, including antigen dose, type of APC and the expression of the mentioned membrane-bound costimulatory signals of the T-helper cells, a so-called Th1-type, and to some degree a Th2-type, response develops during BCG treatment. The Th1 or cell-mediated immune response and the Th2 or humoral immune response are characterised by the patterns of cytokines, secreted by the CD4^+^ T-helper cells following antigen-specific stimulation. In mouse models, the Th1 response is primarily characterised by IL-2, IL-12, IFN-*γ* and TNF-*β*, and the Th2 response by IL-4, IL-5, IL-6 and IL-10. The recognition that this description of two types of responses reflects the human immune response and that they are regulated in a reciprocal fashion, critically regulated by the cytokines, represent a major advance in the field of antitumour cytotoxicity mechanisms.

Knowledge concerning the APC–T-helper cell interaction and development of Th1 and Th2 responses has been used to study the BCG-induced role of the urothelium in antigen presentation and initiation of the immune response.

#### Uroepithelial tumour cells and antigen-presenting properties

Immunocompetence of the host is essential for BCG therapy. Dendritic cells, macrophages and CD4+ T lymphocytes play a crucial role in the antitumour effect. In addition, evidence exists for an important role of epithelial, TCC cancer cells. Mouse bladder tumour cells present BCG antigen to CD4^+^ T cells, via MHC class II molecules (Lattime *et al*, 1992). The initial observations were extended for another murine cell line and a panel of human cell lines. Constitutive and BCG-induced expression of MHC class II and the major costimulatory molecules ICAM-1 and B7-1 was observed. The antigen presentation factors are enhanced in high-grade TCC cell lines only (Figure 1D) (Ikeda *et al*, 2002). Quantitative immunohistochemistry has confirmed the *in vitro* findings. Serial bladder biopsies and urinary cytospins, taken before and after BCG therapy, revealed an upregulation of MHC class II and ICAM-1 expression of urothelial tumour cells (Jackson *et al*, 1994).

Interestingly and likely of uttermost importance, recently non-MHC-encoded, CD1-restricted presentation of (glyco)lipid antigens has been recognised (Maksymowych and Kane, 2000). Although for BCG no data are available yet, CD4^+^ CD1-restricted T cells were observed in patients suffering from *Mycobacterium leprae* (Sieling *et al*, 2000). These cells produce IFN-*γ*, but not IL-4. Bacillus Calmette-Guérin therapy-related research has revealed an increased CD1 expression of TCC cell lines in the presence of live BCG (Ikeda *et al*, 2002) and a virtual absence of urinary IL-4 during BCG treatment (Nadler *et al*, 2003). Accordingly, it is tempting to hypothesise that CD1-facilitated BCG antigen presentation contributes to the development of BCG immunity.

In summary, epithelial TCC cells gain the phenotypical characteristics and functioning of APCs in the presence of BCG. These functions strongly suggest that the BCG–high-grade tumour cell interaction acts in cohort with the BCG–professional APC interaction. Clinical observations seem to confirm this conclusion, since BCG therapy seems to exert a better antitumour effect in high-grade bladder cancer (Table 1).

### Secretion of cytokines by urothelial tumour cells

Multiple studies have reported on cytokine production as a result of BCG therapy. Following intravesical BCG instillations, there is an increase in the urinary level of several cytokines, such as IL-1, IL-2, IL-6, IL-8, IL-12, IL-18, TNF-*α* and IFN-*γ*. Undoubtedly, the major cell sources are the immunocompetent cells, but urothelial cells contribute to a significant degree. *In vitro* studies with human TCC cells revealed a BCG-induced upregulation of the cytokines IL-6, IL-8, IL-10, GM-CSF, TNF-*α* and IFN-*α*, but not IFN-*γ*, IL-2, IL-4 and IL-12 (Figure 1F and G) (Boer de *et al*, 1997; Bevers *et al*, 1998). Of all cytokines produced by TCC cell lines, the cytokines IL-6 and IL-8 are highly prominent and their role will be discussed here in some detail.

#### Interleukin-6

Within the challenged immune system, several cell types including APC produce IL-6. This multifunctional cytokine is critically involved in the acute phase response, T-cell proliferation, B-cell maturation, macrophage maturation and cytotoxic T-cell differentiation. In combination with IL-1, IL-2 and IFN-*γ*, IL-6 induces expression of the IL-2 receptor (IL-2R), thus participating in the activation of resting T cells (Lauta, 2003). Furthermore, Il-6 seems to contribute to MHC-nonrestricted cytotoxic activity by inducing natural killer (NK) cell proliferation. Nowadays, NK cells are considered as essential, cytotoxic effector cells during BCG therapy (Figure 1H) (Brandau *et al*, 2001).

High-grade TCC cell lines show a high constitutive and BCG-induced production of IL-6. However, the importance of IL-6 produced by bladder cancer cells in the BCG-induced immune response remains to be established. *In vitro* studies revealed that IL-6 mRNA upregulation and IL-6 production depend on BCG dose, incubation period, BCG internalisation and TCC cell grade (Bevers *et al*, 1998). Interleukin-6 production requires a minimal BCG exposure time of 0.5–1 h, which is in accordance with clinical practice.

Interleukin-6 is considered a major source of early Th1/Th2 control during CD4^+^ T-cell activation as it attributes to the promotion of the Th2 response and simultaneously inhibition of Th1 polarisation (Figure 1G). Interleukin-6 activates the production of IL-4 by CD4^+^ T cells and their differentiation into Th2 effector cells. Moreover, it inhibits Th1 differentiation by interference with IFN-*γ* signalling and the development of Th1 cells (Diehl and Rincon, 2002). The abundant IL-6 response during BCG therapy and virtually the absence of urinary IL-4 seem to be however in conflict with the recognition that the presence of BCG primarily induces a Th1 response. The absence of IL-4 and a high production of IFN-*γ* may prevent or contradict the Th2-promoting effect of IL-6. Moreover, this finding suggests an important role of the CD1-restricted presentation of (glyco)lipid antigens of *live* BCG , since CD4^+^ CD1-restricted T cells produce IFN-*γ* but not IL-4 (Sieling *et al*, 2000; Ikeda *et al*, 2002).

#### Interleukin-8

Interleukin-8 is a proinflammatory cytokine with strong chemotactic properties, attracting T lymphocytes and neutrophils. Interleukin-8 is induced rapidly, already after the first instillation, during BCG therapy (Boer de *et al*, 1997). Dendritic cells, macrophages and a number of other cells, including TCC cell lines, produce IL-8. The early urinary IL-8 production *in vivo* may indicate the significance of an interaction of BCG bacilli with (residual) bladder cancer cells in the initiation of the host immune response.

### Cell-mediated antitumour effects: the effector cells

The final step in the eradication of tumour cells consists of mobilisation and activation of cytotoxic effector cells (Figure 1H). Several *in vitro* studies show evidence for several nonspecific cytolytic cells like NK cells, BCG-activated killer cells (BAKs), macrophage-activated killer cells (MAKs), lymphokine-activated killer cells (LAKs) and cytotoxic T lymphocytes (Kawashima *et al*, 2003).

A key role is supposed for NK cells (Brandau *et al*, 2001). NK cells, a special population of mononuclear cells, recognise ‘self’-peptides presented by MHC class I molecules on the surface of cells. A cell not displaying these peptides in a correct way is attacked and killed by NK cells (Kärre, 1995). In untreated bladder cancer patients, a loss or alteration of MHC class I expression is seen in tumour cells (Saint *et al*, 2001). Bacillus Calmette-Guérin-infected cells present BCG glycoprotein and lipoprotein antigens on their MHC class I molecule (Neyrolles *et al*, 2001). This may be a trigger for NK cells to attack BCG-infected urothelial tumour cells. Bacillus Calmette-Guérin therapy in the murine model, using NK cell-deficient mice, is ineffective (Brandau *et al*, 2001). However, in studies regarding the presence of NK cells after BCG therapy, relatively few NK cells were seen 3 weeks after the last instillation of a 6-week course (Lattime *et al*, 1992; Saint *et al*, 2001). It would be interesting to know if NK cells are more abundant earlier, during the BCG course. Strong direct evidence for NK cell or other effector cell(s) is still lacking. The recent acknowledgement of effector cells that recognise mycobacterial (glyco)lipid antigens through nonpolymorphic MHC molecules, such as CD1, may provide new insights into the true nature of the cytolytic effector cells involved in tumour cell eradication during BCG treatment (Maksymowych and Kane, 2000; Kawashima *et al*, 2003).

## SUMMARY AND CONCLUSIONS

Current insight of the mode of action of BCG, ranging from its introduction into the bladder to killing of residual tumour cells, has revealed a complex sequence of processes. Bacillus Calmette-Guérin accumulates near, and adheres to, the bladder wall. After passage through the GAG layer, BCG is internalised and processed by professional APC and tumour cells. The modified gene expression of these cells accumulates in the secretion of particular cytokines and presentation of BCG antigens. Bacillus Calmette-Guérin antigens are presented via MHC class II molecules to CD4^+^ T cells and via MHC class I molecules to CD8^+^ T cells. Lipid and glycolipid BCG antigens can be presented to CD4^+^ and CD8^+^ T cells in a non-MHC-restricted, CD1-restricted fashion. Production of chemokines, such as IL-8, secreted partly by BCG-internalised tumour cells, contributes to the local activation of the immune system. Consequently, activated leucocytes and mononuclear cells invade the bladder wall. These developments provide the condition for a Th1 response, associated with particular cytokines (IFN-*γ*, IL-2, IL-12 and TNF-*β*). This cytokines profile promotes delayed-type hypersensitivity response, cytotoxic cell response, and macrophage activation or cellular immune inflammatory reaction. Depending on bacterial and host components, an upregulation of the Th2 response, associated with cytokines IL-6 and IL-10, may occur to some degree and adversely affect the functioning of the Th1 response. The Th1 cytokine profile enables recruitment and maturation of cytotoxic effector cells.

No definite statements can be made yet about the actual effector cell(s), but a crucial, cytotoxic role of NK cells has been proposed. In addition, some of the cytokines, and BCG itself, may exhibit a direct cytotoxic effect on tumour cells.

In 28 years of major research efforts, understanding of the mode of action underlying BCG therapy for bladder carcinoma is obviously much improved. Yet the jigsaw is not complete and many details wait unraveling. However, if successful, the reward might be a better, evidence-based BCG immunotherapy with optimal clinical efficacy and minimal occurrence of side effects in the form of an optimal BCG dose and treatment schedule, genetically engineered BCG, or particular antigenic molecule(s) that trigger immunological antitumour activity in a well-controlled manner.
